# Supplementary material for: The interactome of intact mitochondria by cross-linking mass spectrometry provides evidence for coexisting respiratory supercomplexes
Source: Mol Cell Proteomics. 2017 Dec 8;17(2):216–32. doi: 10.1074/mcp.RA117.000470 (PMC5795388; doi:10.1074/mcp.RA117.000470)
Supplement: Supplemental Data [file supp_RA117.000470_133922_0_supp_23978_fzffwf.zip › spectra_annotation/mito_spectra_annotation/494-1-6-1-12-1.pdf]

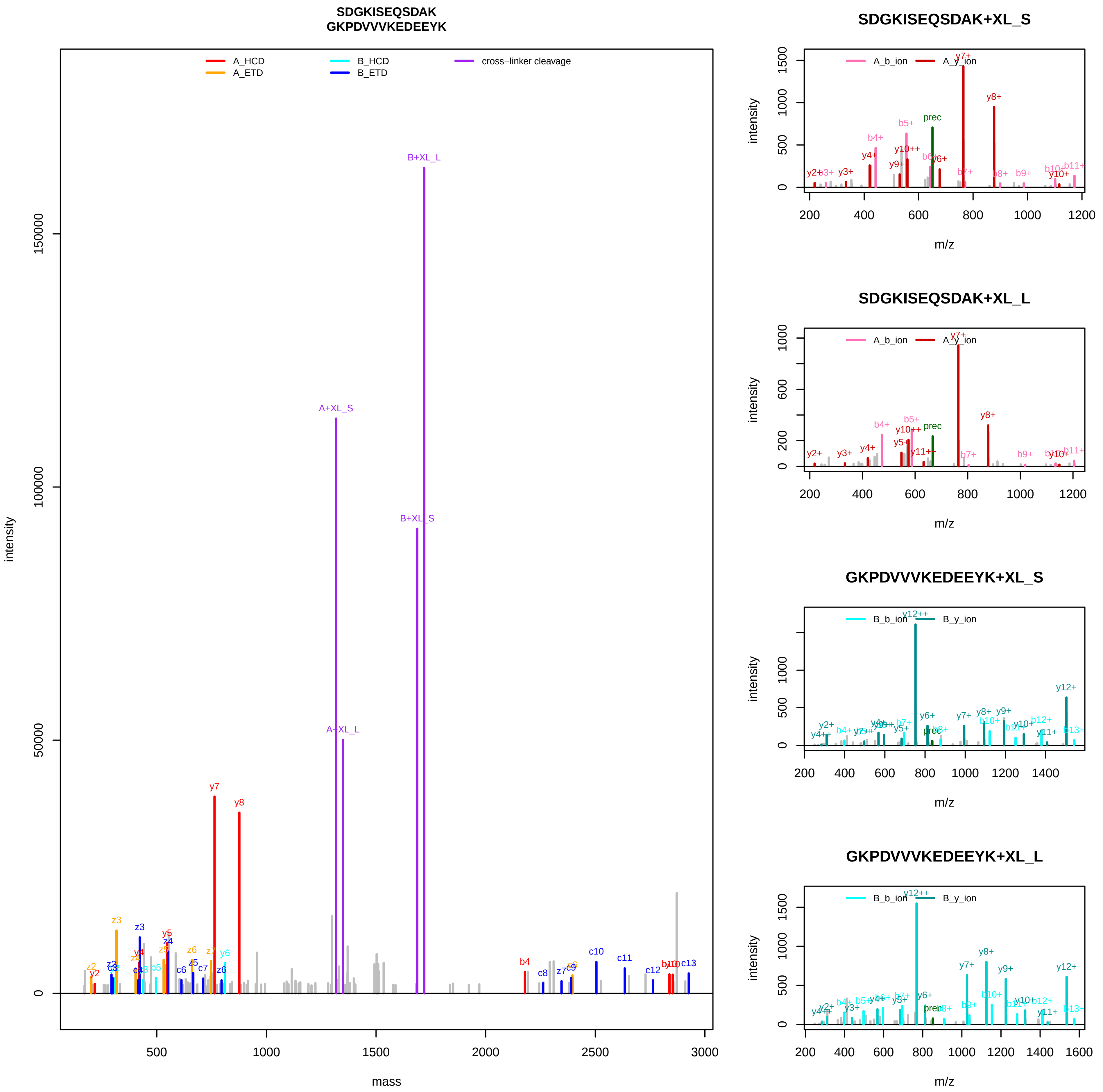
SDGKISEQSDAK
GKPDVVVKEDEEYK
A_HCD
A_ETD
B_HCD
B_ETD
cross−linker cleavage
B+XL_L
A+XL_S
B+XL_S
A+XL_L
y7
y8
z3
z3
y5
z4
y4
b4
c8
z7
c9
c10
c11
c12
b10
c13
intensity
mass
0
50000
100000
150000
500
1000
1500
2000
2500
3000
SDGKISEQSDAK+XL_S
A_b_ion
A_y_ion
y7+
y8+
prec
b5+
b4+
y10++
y4+
y9++
y6+
y2+
b3+
y3+
b7+
b8+
b9+
b10+
b11+
y10+
intensity
m/z
SDGKISEQSDAK+XL_L
A_b_ion
A_y_ion
y7+
y8+
b5+
b4+
y10++
prec
y5+
y4+
y11++
y2+
y3+
b7+
b9+
y10+
b11+
intensity
m/z
GKPDVVVKEDEEYK+XL_S
B_b_ion
B_y_ion
y12++
y12+
y6+
y7+
y8+
y9+
b10+
y10+
b12+
y11+
b13+
y2+
y4++
b4+
y3+
b7+
y5+
prec
b8+
intensity
m/z
GKPDVVVKEDEEYK+XL_L
B_b_ion
B_y_ion
y12++
y8+
y7+
y9+
y12+
y6+
b10+
y10+
b12+
y11+
b13+
y2+
y4++
b4+
y3+
b5+
b7+
y5+
prec
b8+
b9+
intensity
m/z
